# Grazing preference and isotopic contributions of kelp to *Zostera marina* mesograzers

**DOI:** 10.3389/fpls.2022.991744

**Published:** 2022-10-13

**Authors:** Angeleen M. Olson, Carolyn Prentice, Zachary L. Monteith, Derek VanMaanen, Francis Juanes, Margot Hessing-Lewis

**Affiliations:** ^1^ Nearshore Ecology, Hakai Institute, Heriot Bay, BC, Canada; ^2^ Fisheries Ecology and Conservation Lab, Department of Biology, University of Victoria, Victoria, BC, Canada; ^3^ Institute for the Oceans and Fisheries, University of British Columbia, Vancouver, BC, Canada

**Keywords:** seascape, epiphytes, stable isotopes, allochthonous subsidies, trophic interactions, eelgrass

## Abstract

In seagrass food webs, small invertebrate mesograzers often exert top-down control on algal epiphytes growing on seagrass blades, which in turn releases the seagrass from competition for light and nutrients. Yet, nearshore habitat boundaries are permeable, and allochthonous subsidies can provide alternative food sources to *in-situ* production in seagrass meadows, which may in turn alter mesograzer-epiphyte interactions. We examined the contribution of allochthonous kelp (*Nereocystis luetkeana*), autochthonous epiphytic macroalgal (*Smithora naiadum*), *Ulva lactuca*, and seagrass production to mesograzer diets in a subtidal *Zostera marina* (eelgrass) meadow. In both choice feeding experiments and isotopic analysis, mesograzer diets revealed a preference for allochthonous *N. luetkeana* over *Z. marina*, *S. naiadum*, and *U. lactuca*. Notably, *Idotea resecata* showed an ~20x greater consumption rate for *N. luetkeana* in feeding experiments over other macrophytes. In the meadow, we found a positive relationship between epiphytic *S. naiadum* and gammarid amphipod biomass suggesting weak top-down control on the *S. naiadum* biomass. Epiphyte biomass may be driven by bottom-up factors such as environmental conditions, or the availability and preference of allochthonous kelp, though further work is needed to disentangle these interactions. Additionally, we found that gammarid and caprellid amphipod biomass were positively influenced by adjacency to kelp at seagrass meadow edges. Our findings suggest that *N. luetkeana* kelp subsidies are important to the diets of mesograzers in *Z. marina* meadows. Spatial planning and management of marine areas should consider trophic linkages between kelp and eelgrass habitats as a critical seascape feature if the goal is to conserve nearshore food web structure and function.

## 1 Introduction

The recognition that ecosystems are connected across boundaries by nutrient and organism flow has broadened our understanding of trophic interactions within and among ecosystems (Loreau et al., 2003; Marczak et al., 2007). Energy that cross ecosystem boundaries - known as allochthonous subsidies - can play key roles in population and community structure of recipient habitats, and in turn, the function and stability of these ecosystems (Polis and Hurd, 1996; Huxel et al., 2002). The importance of allochthonous subsidies is now recognized in nearly all aquatic and terrestrial ecosystems (Lafage et al., 2019), from tropical and temperate rainforest insect communities (Recalde et al., 2016; Recalde et al., 2020; Nakano and Murakami, 2001) to freshwater planktonic communities (Vargas et al., 2011, Adamczuk et al., 2019), subtropical island ecosystems (Spiller et al., 2010) and coastal marine ecosystems (Savage, 2019; Zuercher and Galloway, 2019). While allochthonous subsidies are often overlooked when assessing ecosystem interactions (Buckner et al., 2018; Smale et al., 2018), they are increasingly recognized as having important influences on the composition of food web producers, as well as trophic transfer through food webs.

The availability of allochthonous foods can alter interactions between primary producers and consumers in the recipient ecosystem (Huxel and McCann, 1998; Huxel et al., 2002). For instance, if allochthonous inputs weaken specific autochthonous producer - consumer interactions, then expected trophic cascades could be dampened (Polis and Hurd, 1996; Huxel and McCann, 1998; Rodewald et al., 2011). The influence of an allochthonous subsidy may depend on the characteristics of the trophic subsidy itself (e.g. its duration, timing, spatial extent, palatability) as well as characteristics regulating consumers in the recipient habitat, such as availability of other food sources, consumer size, feeding mode, trophic level and life history stage (Zuercher and Galloway, 2019). Previous work has largely focused on the effect of cross-ecosystem subsidies on nutrient-poor recipient ecosystems such as desert islands (Anderson and Polis, 1999) and sandy beaches (Lastra et al., 2008; Liebowitz et al., 2016), as well as aquatic subsidies into riparian ecosystems (Hocking and Reimchen, 2009; Lafage et al., 2019). Growing evidence suggests cross-ecosystem energy transfer may be important to highly productive recipient ecosystems, such as mangroves (Slim et al., 1996), coral reefs (Carreón-Palau et al., 2013) and seagrass meadows (Hyndes et al., 2012, Cartraud et al., 2021).

In seagrass ecosystems, a central tenet of food web structuring is the top-down role of small invertebrate herbivores (herein ‘mesograzers’) in consuming algal epiphytes, which releases seagrass from negative impacts of algal shading and/or nutrient competition and maintains a seagrass-dominated ecosystem (Orth and Van Montfrans, 1984; Valentine and Duffy, 2006; Cook et al., 2011). Empirical evidence for this indirect positive effect of mesograzers on seagrass productivity has been demonstrated thoroughly (Orth and Van Montfrans, 1984; Hughes et al., 2004; Moksnes et al., 2008; Whalen et al., 2013) and is increasingly important to our understanding of bottom-up and top-down human disturbances to seagrass meadows (e.g., eutrophication and overfishing, respectively). Although negative relationships between mesograzers and epiphytes are widely generalized, their strength and direction can depend on seasonality (Whalen et al., 2013), the source of nutrient inputs (Hessing-Lewis and Hacker, 2013), mesograzer species composition (Duffy and Harvilicz, 2001; Jaschinski and Sommer, 2008), and predation rates on mesograzers (Moksnes et al., 2008; Hughes et al., 2013).

Seagrass habitats have high *in-situ* productivity and are known to contribute significant amounts of biomass to adjacent ecosystems such as the deep sea and sandy beaches (Heck et al., 2008; Liebowitz et al., 2016; Duarte and Krause-Jensen, 2017). Yet, their role as recipients of allochthonous materials has received relatively less attention. Seagrass meadows often occur in sheltered coastal environments, where their canopies facilitate the deposition of materials from the water column, leading to enhanced accumulation of allochthonous materials (Peterson et al., 2004; Hendriks et al., 2008). Among many potential allochthonous inputs, the role of kelp is emerging as an important food source for organisms in temperate seagrass meadows (Smit et al., 2006; Hyndes et al., 2012; Hyndes et al., 2014; Olson et al., 2019). For example, along the Atlantic coast of Canada, an estimated 82% of annual kelp productivity enters detrital pathways, which can enhance secondary production in recipient food webs (Krumhansl and Scheibling, 2012). Accumulation of kelp biomass in seagrass meadows can be substantial with transportation to meadows occurring from considerable distances away (Wernberg et al., 2006). However, the extent to which kelp may be incorporated into recipient seagrass food webs, and if they can alter mesograzer-producer interactions, remains unclear.

In this study, we examined the contribution of allochthonous bull kelp (*Nereocystis luetkeana*) to the diets of mesograzers in a temperate seagrass *Zostera marina* (common name ‘eelgrass’*)* relative to other ubiquitous macrophytes. We quantified the preference for allochthonous vs. autochthonous sources to mesograzers using choice feeding experiments. Next, we assessed the contributions of these same macrophytes to *in-situ* mesograzer diets using natural isotopic tracers. Because of the widespread presence of kelp forests in this seascape, and their high nutrient quality, we expected kelp subsidies to make up a significant proportion of mesograzer diets. Finally, kelp subsidies may mediate mesograzer – macrophyte interactions. As a first step in assessing the trophic influence of kelp on the *Z. marina* food web (see Hessing-Lewis et al., 2018), we examined relationships between common mesograzers and a dominant *Z. marina* epiphyte (*Smithora naiadum*), as well as the effect of meadow location on mesograzer biomass.

## 2 Methods

**Figure 1:**
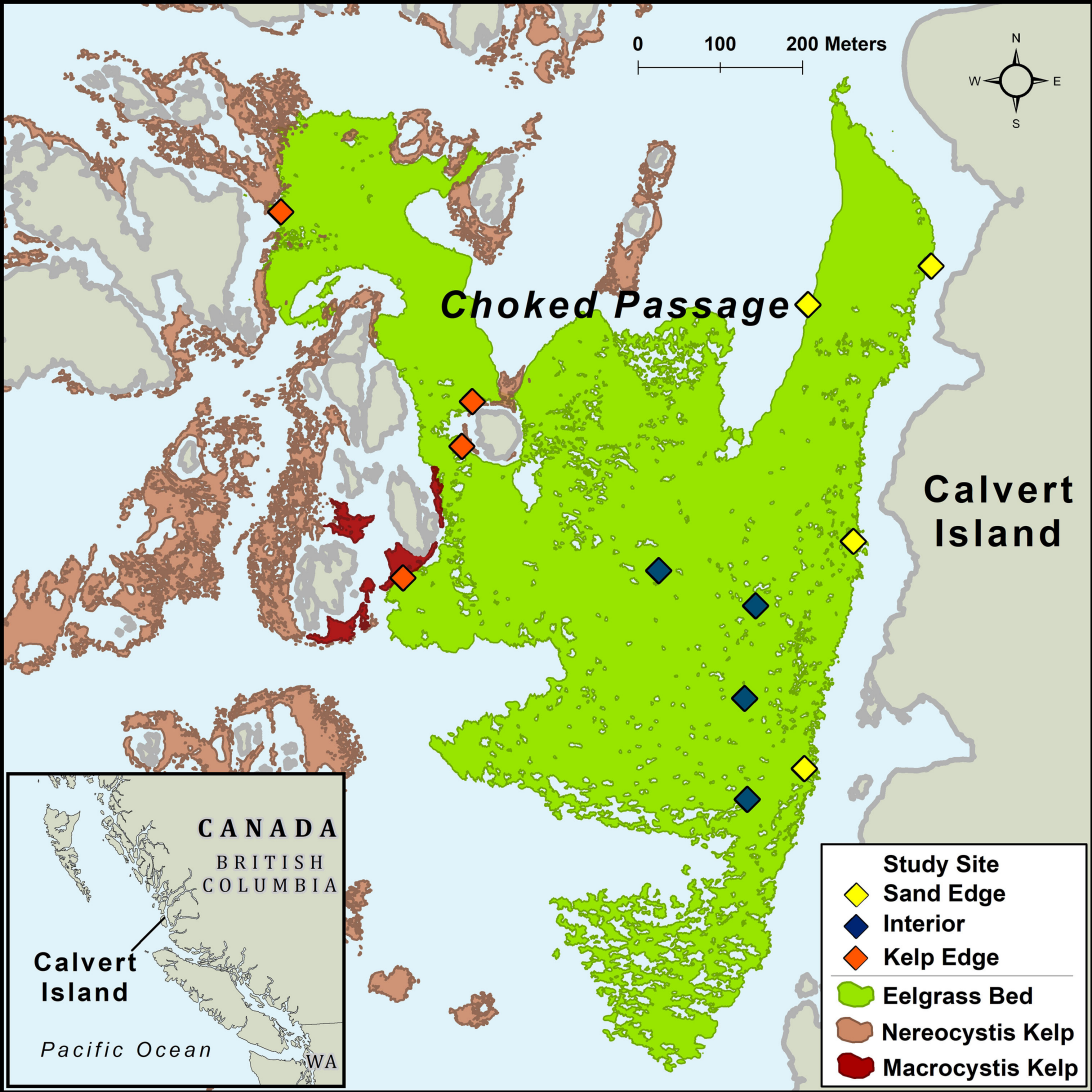
Study area off Calvert Island, British Columbia, Canada. Sites were established in a *Zostera marina* meadow in Choked Passage (green) which is located in a nearshore seascape surrounded by canopy forming kelp, primarily *Nereocystis luetkeana* (light red) in proximity to some *Macrocystis pyrifera* (dark red).

British Columbia’s (B.C.) coastline is characterized by high complexity, including exposed outer-coast islands, sheltered bays, estuaries, and steep fjords. On the central coast of B.C., the nearshore environment is a heterogenous seascape consisting of kelp forests, seagrass meadows, benthic algae, rocky reefs, and sandy habitats. Our study was conducted in a large subtidal *Z. marina* meadow located in Choked Passage on the northern shore of Calvert Island in the summer of 2015 (**Figure 1**). This *Z. marina* meadow is predominantly surrounded by shallow rocky reefs, bare sand habitats, annual *N. luetkeana* kelp forests, and to a lesser extent perennial *Macrocystis pyrifera* kelp forests.

### 2.1 Choice feeding experiments

In August 2015, we conducted a multiple-choice feeding experiment with mesograzers collected from the Choked Passage meadow to determine their dietary preference among allochthonous (*N. luetkeana*) and autochthonous (*Z. marina, S. naiadum*) macrophytes, as well as *Ulva lactuca* (both allochthonous and autochthonous). We focused on mesograzers with larger body size because of their ubiquity in local *Z. marina* meadows and adjacent kelp forests and ease to work with. Mesograzers and macrophytes were collected haphazardly from the meadow, focusing on isopods, *I. resecata*, (mean 23 ± 4 mm length (SD) and 116.3 ± 33.6 mg biomass in our study) and cryptic kelp crabs, *Pugettia richii*, a larger body size grazer (max. size 44 mm, Lamb and Hanby, 2005) averaging 5962.9 ± 4421.9 mg in our study.

The experiment took place in a natural flow-through seawater system. Mesograzers were starved for 35 hours prior to the feeding experiments, weighed, and placed in separate small containers with fine mesh windows open to seawater off the Hakai Institute Observatory dock in Pruth Bay directly adjacent to another *Z. marina* meadow. For the experiment, mesograzers were placed in an experimental container (946 mL volume) which included four macrophytes of equal surface area (2 x 2 cm square): *Z. marina, N. luetkeana*, *S. naiadum* and *U. lactuca*. The containers were suspended off the dock and subject to natural daylight patterns and temperatures (**Supplementary Figure 1**). Replicate trials were conducted for each mesograzer type along with simultaneous control trials with no mesograzers present (n = 7). Experimental containers were otherwise bare (e.g., no sand or habitat substitutes). Macrophytes were blotted dry and weighed on a microbalance (mg) before and after the duration of the feeding trials, which were run for 18 hours. Macrophyte consumption rates by each mesograzer were calculated using Equation 1 (Taylor and Brown, 2006; Sampaio et al., 2017), which accounted for consumer size (i.e., biomass in mg) and the change in the weight of the macrophyte relative to a grazer-free control:


(1) $$Consumption\;Rate\;=\;\frac{Ti\;*\;(Cf/Ci)-\;Tf}{n\;bio\;*\;t}$$

where T_i_ is the initial producer blotted wet weight (bww), T_f_ is the final bww, C_i_ is the initial control bww, C_f_ is the final control bww, n_bio_ is the grazer biomass (i.e., size) at the end of the experiment (g), and t = duration of the experiment in days. Consumption rate is thus expressed in mg of macrophyte consumed per mesograzer biomass per day (mg PP/mg grazer/day). A one-way analysis of variance (ANOVA) was used to test for differences in the consumption rates for both *I. resecata* and *P. richii* on the four macrophytes. If significant differences were detected (P< 0.05), a Tukey’s *post-hoc* test was used to examine pairwise comparisons amongst all macrophytes used in the feeding trials.

### 2.2 Stable isotopes and mixing models

We collected mesograzers from *Z. marina* shoots during a 2-week period in late July- early August 2015 from the kelp edge, sand edge, and interior sites. Mesograzers were frozen until laboratory processing for isotope analysis. Due to their small body sizes, numerous individuals of gammarid amphipods (multiple species in the family Gammaridae) and Lacuna snails (multiple species of the *Lacuna* genus,) were pooled within a single sample (n = 10 individuals/sample, 5 samples total). Other mesograzers were large enough that an individual’s biomass filled a sample: Caprellid amphipods (multiple species of the Caprellidae family, n = 13), *I. resecata* (n = 13), and *P. richii* (n = 3). Isotope signatures of most macrophytes were obtained from a concurrent study (see Olson et al., 2019), which included *Z. marina* and *S. naiadum* as *in-situ* meadow production and *N. luetkeana* from surrounding kelp forests. For this study, *U. lactuca* (n = 20) was also collected adrift in the *Z. marina* meadow. Small amounts of *U. lactuca* were found growing in the seagrass meadow and also was commonly found growing in the surrounding reef or sand habitats rather than within the meadow (authors’ personal obs.), and thus could be considered as both autochthonous and/or allochthonous production.

Samples were prepared for isotope analysis by defrosting and removing surface debris. Lacuna snail bodies were pulled out of their shells for processing. Whole bodies of the other mesograzers (including stomachs) were rinsed in two baths of deionized water and dried at 60°C. δ^13^C and δ^15^N were analyzed at the Mazumder Lab at the University of Victoria on a Delta IV Isotope Ratio Mass Spectrometer, as the ratio of heavy to light isotope with values denoted in *δ*:


(2) $$\delta \left(‰\right)=\left(\frac{\left(Rsample\right)}{\left(Rstandard\right)}-1\right)\;\times 1000$$

where R represents the ratios 13C/12C and 15N/14N of the sample or laboratory standard. Mass ratios of carbon to nitrogen (C:N) were also determined for macrophytes, and were used to assess their relative palatability, where lower values of C:N represent relatively higher nutritional content. An ANOVA test was used to assess differences in the C:N among macrophytes, with a Tukey’s *post-hoc* test to further contrast between each combination.

We used a Bayesian isotopic mixing model mixSIAR (Moore and Semmens, 2008, Stock et al., 2018) to assess the relative contribution of the macrophytes to mesograzer diets. When predators consume prey energy, the heavy isotope is favoured over the light isotope due to discrimination from metabolic processes, which causes an enrichment of isotope values with trophic level. Thus diet–tissue discrimination factors (DTDFs) are used when estimating prey contribution to a predator’s diet. Because mixing model results are highly sensitive to DTDFs, we assessed two options from the literature: 0.4 ± 0.12‰ for δ^13^C and 2.0 ± 0.20‰ for δ^15^N (McCutchan et al., 2003) and 0.4 ± 1.14‰ for δ^13^C and 3.4 ± 1.0‰ for δ^15^N (Post, 2002). After visual assessment of the consumer isotopic values alongside macrophyte values post correction (Phillips et al., 2014), DTDFs from Post et al. (2002) were chosen because of their better fit (i.e., consumer values fell within the range of macrophyte mean and standard deviation values). During this analysis, one caprellid amphipod outlier with depleted δ^13^C values outside the macrophyte isotope ranges was removed.

### 2.3 Establishing mesograzer-epiphyte relationships

To assess biomass and abundance of macrophytes and mesograzers, twelve sites were established in the *Z. marina* meadow (total area ~367,000m^2^; **Figure 1**). 40 m transects were set in the *Z. marina* meadow adjacent to *N. luetkeana* kelp forests (n=4 transects), adjacent to sand habitats (n=4), and in the interior of the meadow (n=4) at depths that ranged from 1.52m – 4.99m. We collected *Z. marina* shoots at 10 m intervals (n = 5 shoots per transect) by scuba. Divers carefully covered shoots with a plastic bag, detached the shoot from the rhizome, and sealed the bag before moving to the next collection point. Sampling occurred in May, July, and August 2015. Shoot samples were subsequently processed in the laboratory. Bag contents were sieved through a 500um filter, capturing mesograzers > 500um. *Z. marina* shoots were gently scraped of epiphytes and mesograzers. All components were oven dried at 60°C for biomass measurements.

We used a binomial - gamma hurdle generalized linear model (GLM) to assess the relationship between mesograzer biomass and *S. naiadum* biomass in R (R Core Team, 2021). Mesograzers and *S. naiadum* dry biomass (g) were standardized by dry *Z. marina* blade biomass (g) from which they were collected. We first modeled the probability of presence or absence of each mesograzer’s biomass related to *S. naiadum* biomass with a binomial distribution. A gamma model was then used on non-zero grazer biomass to estimate the relationship between grazers and *S. naiadum* biomass. If residual plots indicated high leverage points in preliminary model analysis, these data were removed by a Cook’s Distance cut off.

## 3 Results

### 3.1 Feeding experiment results: Mesograzer preferences

**Figure 2:**
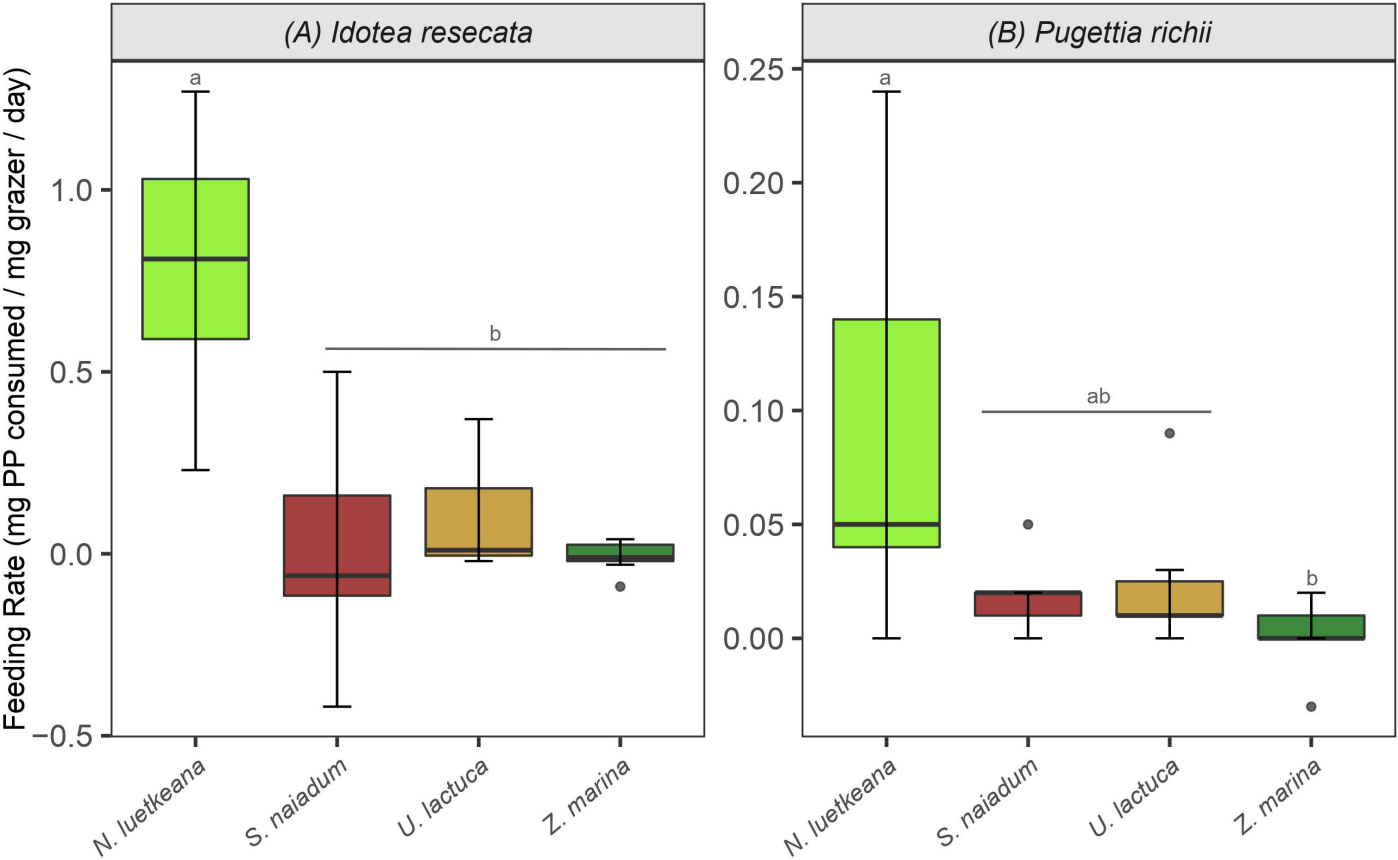
Consumption rates (mean and standard error) by **(A)** *I. resecata* isopods and **(B)** *P. richii* crabs on macrophytes from the feeding trials - N*. luetkeana*, *S. naiadum*, *U. lactuca* and *Z. marina*. Note the differing scales for each mesograzer. Letters indicate treatments that are significantly different from one another. The figure represents the full and raw dataset, and negative values derived from the consumption equation were retained for completeness.

*I. resecata* and *P. richii* consumed all four macrophytes provided to them (*N. luetkeana*, *U. lactuca*, *Z. marina* and *S. naiadum*) to some extent. Control replicates (no grazers present) showed little loss of biomass and any changes that did occur were accounted for in consumption rate calculations (Equation 1). Preferences for macroalgae over *Z. marina* were demonstrated by both mesograzers, as well as a notably high consumption rate of *N. luetkeana* kelp by *I. resecata* isopods - 0.8 mg kelp/mg grazer/day (**Figure 2A**).

*I. resecata* consumed the four macrophytes at different rates (**Figure 2A**, ANOVA: F_(3, 24)_ = 17.21, P< 0.001). Specifically, they consumed *N. luetkeana* at a greater rate than *U. lactuca*, *S. naiadum* and *Z. marina* (P< 0.001 for all pairwise comparisons); consumption rates were ~20x higher for *N. luetkeana* relative to the other three options. Further, there was no difference in their consumption rate of each combination of *S. naiadum*, *U. lactuca* and *Z. marina* (**Figure 2A**). Similar to *I. resecata*, *P. richii* consumed macrophytes at different rates (**Figure 2B**, ANOVA: F_(3, 24)_ = 4.271, P = 0.015). The largest difference in consumption rate by *P. richii* was observed between *N. luetkeana* and *Z. marina* (P = 0.014). No other pairwise comparisons of consumption rates for *P. richii* were significantly different (**Figure 2B**).

### 3.2 Isotopic results and feeding observations from the field

**Figure 3:**
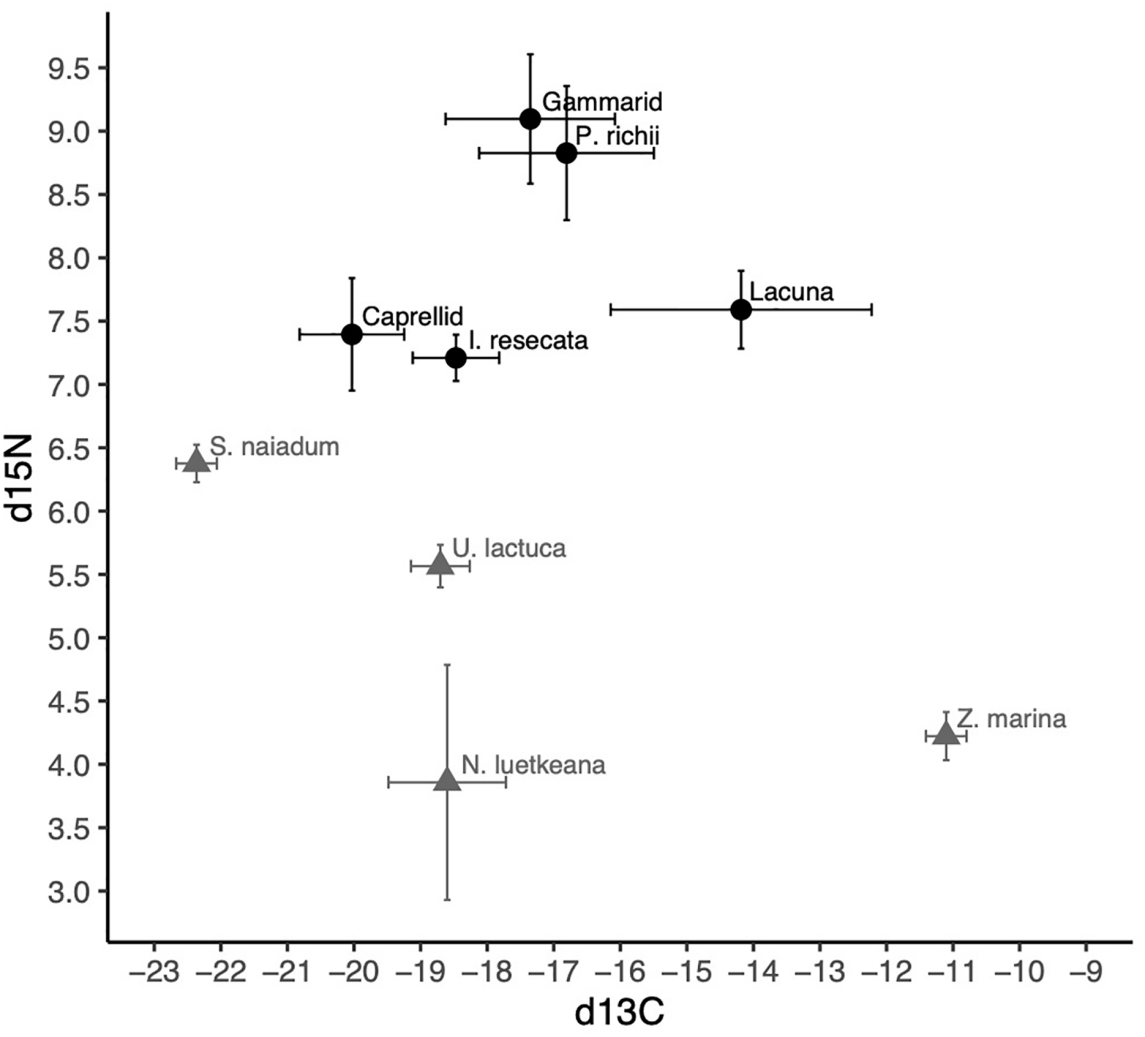
δ^13^C and δ^15^N signatures (mean and standard deviation) of invertebrate mesograzers (black) and macrophytes (grey) in the Choked Pass *Z. marina* meadow.

The isotopic composition of mesograzer in the *Z. marina* meadow was variable particularly in δ^13^C (**Figure 3**). Caprellid amphipods had the most depleted δ^13^C signatures, while Lacuna snails were most enriched. δ^15^N signatures of the mesograzers were much closer in range, where gammarid amphipods and *P. richii* were more enriched relative to *I. resecata*, caprellid amphipods, and Lacuna snails (**Figure 3**). δ^13^C and δ^15^N values of *U. lactuca* fell in between *Z. marina* (most enriched) and *S. naiadum* (most depleted). *N. luetkeana*, *S. naiadum*, and *U. lactuca* macroalgae had lower C:N ratios than *Z. marina* (pairwise comparisons, P<0.001). Macrophytes differed in palatibility as measured by C:N ratios (**Table 1**, ANOVA: F_(3,63)_ = 158.1, P< 0.001). *S. naiadum* had the lowest C:N ratios of all macrophytes examined (P<0.05), specifically ~ 3x lower than the other autochthonous macrophyte, *Z. marina*. We found no difference between *N. luetkeana* and *U. lactuca* C:N ratios (P = 0.29).

**Table 1:** Macrophyte carbon to nitrogen ratios (C:N) in the *Z. marina* meadow indicating their relative palatability.

Macrophyte	Mean C:N	SD C:N
*Nereocystis luetkeana*	10.61	0.44
*Zostera marina*	18.16	2.34
*Smithora naiadum*	7.40	0.93
*Ulva lactuca*	9.03	1.66

Lower C:N values indicate higher palatability.

**Figure 4:**
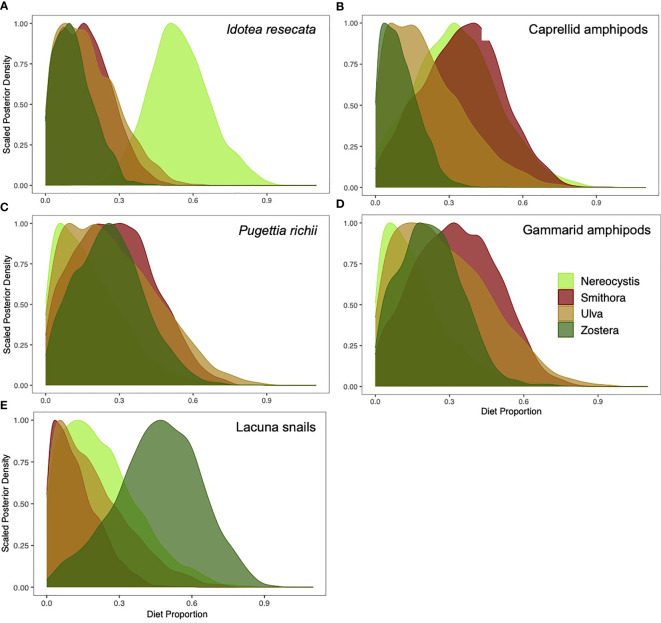
Proportion of *N. luetkeana*, *S. naiadum, U. lactuca*, and *Z. marina* that contributed to mesograzers diets: **(A)** *I*. *resecata*, **(B)** caprellid amphipods **(C)** *P. richii* crabs, **(D)** gammarid amphipods, and **(E)** Lacuna snails.

Contributions of allochthonous vs. autochthonous macrophytes to diets varied by mesograzer (**Figure 4**). Allochthonous sources were high in *I. resecata* and caprellid amphipods, whereas autochthonous sources contributed more to *P. richii*, gammarid amphipod, and Lacuna snail diets. In *I. resecata* diets, *N. luetkeana* had the highest contribution (54.3%, **Figure 4A**), followed by *U. lactuca* (17.6%) and *S. naiadum* (16.3%). *Z. marina* had the lowest contribution to *I. resecata* diets (12%). For caprellid amphipod diets (**Figure 4B**), *S. naiadum* (35.2%) and *N. luetkeana* (33.6%) showed the highest contributions, whereas *U. lactuca* (21.3%) and *Z. marina* (10.4%) had low dietary contributions.

Autochthonous meadow sources dominated *P. richii* diets: *S. naiadum* (28.1%) and *Z. marina* (26.1%) (**Figure 4C**). Contributions from *U. lactuca* (27.4%) were also high, while contributions from *N. luetkeana* were lowest (18.4%). Similarly, gammarid amphipods diets (**Figure 4D**) had highest contributions from *S. naiadum* (32.7%) followed by *U. lactuca* (28%), *Z. marina* (22.5%) and the lowest by *N. luetkeana* (16.7%). Lacuna snail diets consisted primarily of *Z. marina* (46%), with lower contributions of *N. luetkeana* (22.6%), *U. lactuca* (19.1%), and *S. naiadum* (12.4%).

The relative uptake of primary production by mesograzers did not consistently mirror their relative palatability as assessed by C:N ratio (**Table 1**, **Figure 4**). Caprellid amphipods were the only mesograzer that closely matched their diets to palatability *via* C:N ratio. *S. naiadum* was favoured by most mesograzers (**Figures 4B–D**). *N. luetkeana* contributed more than expected (based on C:N) to *I. resecata* and Caprellids amphipods. *Z. marina* also had higher than expected contributions, as seen in Lacuna snails and *P. richii* crabs (**Figures 4E, C**, respectively).

### 3.3 Field observations of the grazer-producer biomass relationship

The sub-tidal *Z. marina* shoots weighed on average 2.3 ± 0.95 g (SD, dry weight, n = 178), and were characterized as long (146.5 ± 36.0 cm) and wide (0.85 ± 0.15 cm) from n = 115 intact longest blades. The dominant epiphyte across the meadow was the red alga *S. naiadum*. Lobed blades of *S. naiadum* growing from encrusted basal cushions were extensive across the meadow: present on 66% of the blades with an average biomass of 0.82 ± 1.1 g shoot^-1^ up to a maximum value of 5.1 g shoot^-1^. *Punctaria* spp. and *Ulva* spp. epiphytes were present but less abundant - when present, they had minimal biomass on blades (mean of 0.20 ± 0.16 g shoot^-1^ and 0.07 ± 0.05 g shoot^-1^, respectively).

**Figure 5:**
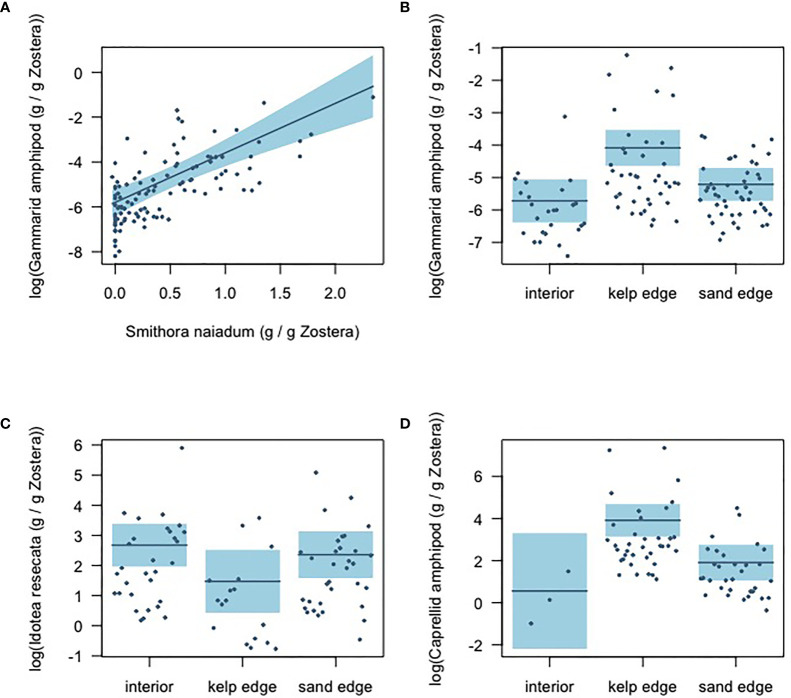
Gamma-hurdle model results showing the **(A)** relationship between *S. naiadum* epiphyte biomass and gammarid amphipod biomass (standardized by *Z. marina* biomass); and the mesograzer biomass distributions across the meadow sites: **(B)** gammarid amphipods **(C)** *I. resecata*, and **(D)** caprellid amphipods. Shaded blue indicates 95% confidence intervals.

Results from the gamma hurdle model indicate that gammarid amphipods and *S. naiadum* biomass had a positive relationship (GLM intercept = -6.30 ± 0.25; *b* = 2.21 ± 0.28, P<0.001) in the eelgrass meadow (**Figure 5A**). The other mesograzers examined did not demonstrate a significant relationship with *S. naiadum* (**Supplementary Figure 2**). The biomass distribution of mesograzers was uneven across the meadow (**Figures 5B–D**). Gammarid and caprellid amphipods had significantly higher biomass at the kelp edge relative to the sand edges and interior sites (GLM *b* = 1.63 ± 0.35, P<0.001, *b* = 3.35 ± 1.11, P =0.004, respectively). In contrast, *I. resecata* biomass was significantly lower at kelp edges than the interior and sand sites (P = 0.020). Lacuna snail biomass was consistent throughout the meadow (**Supplementary Figure 2D**).

## 4 Discussion

### 4.1 Overall findings

Using choice feeding experiments and stable isotopes, we found that mesograzers in a *Z. marina* meadow were consuming allochthonous kelp. Kelp was the preferred food in experiments involving *I. resecata* and *P. richii* mesograzers relative to other primary producers (*S. naiadum, Z. marina, U. lactuca*). Yet, stable isotope results revealed a more varied uptake of allochthonous vs. autochthonous food depending on the mesograzer. *N. luetkeana* was an important contribution to the diets of *I. resecata*, Caprellid amphipods, and Lacuna snails, but less so to *P. richii* and gammarid amphipod diets. Epiphytic *S. naiadum* was the most consistent *in-situ* meadow macrophyte source isotopically integrated into mesograzer diets, highlighting the importance of this epiphyte to the *Z. marina* food web. *S. naiadum* also had the highest palatability *via* C:N ratio which may explain its favourability. We observed a positive relationship between gammarid amphipods and *S. naiadum* epiphytes, suggesting that there may be weak top-down control on epiphyte abundance by grazers in this system (see food web structure in Hessing-Lewis et al., 2018). We hypothesize that bottom-up drivers may play a key role in structuring this *Z. marina* food web, however experiments controlling for potential bottom-up (e.g., currents, allochthonous subsidies, edge effects) and top-down factors (e.g., grazing pressures, species composition) are needed to disentangle those complex interactions in this ecosystem.

### 4.2 Mesograzer feeding preferences and why kelp may be preferentially consumed

While our results suggest the incorporation of kelp into mesograzer diets for some species, we also observed high variability in diets among mesograzer species, as has been found elsewhere (Duffy and Harvilicz, 2001; Douglass et al., 2011). Mesograzers often have the choice of a variety of food sources (e.g., eelgrass, periphyton, bladed epiphytes, detritus/drift algae) that vary in availability and palatability through time and space. Different food sources may be more or less available to mesograzers depending on their mode of feeding (e.g., filter feeders vs. grazing invertebrates). For *I. resecata*, a common eelgrass-dwelling mesograzer, the feeding trial and isotopic data aligned well and suggested a strong preference for *N. luetkeana* over autochthonous *Z. marina* and *S. naiadum* (**Figures 2**, **4A**). This result makes sense as *I. resecata* are highly mobile and able to consume detritus and larger plant material, and are likely to feed on kelp when available. *I. resecata* is known to consume *Z. marina* and microalgae (Best and Stachowicz, 2012; Lewis and Boyer, 2014). Less is known about *I. resecata* feeding preferences for *N. luetkeana*, however they are a well-acknowledged grazer in *M. pyrifera* kelp forests (Ng and Micheli, 2020). Feeding trial results for *P. richii* suggest a strong preference for *N. luetkeana* (**Figure 2**). However, the isotope results suggested a different longer-term trend, with larger contributions of *S. naiadum*, *U. lactuca* and *Z. marina* than *N. luetkeana* (**Figure 4C**).

Mesograzer preference for *N. luetkeana* over autochthonous primary production in *Z. marina* meadows may be explained by its bioavailability and palatability. Although both mesograzer species used for the feeding trials (*I. resecata* and *P. richii*) showed a strong preference for kelp in the lab, *N. luetkeana* subsidies may be temporally and spatially limited in the *Z. marina* meadow. *P. richii* crabs are often in the eelgrass canopy, where access to *Z. marina*, *S. naiadum*, and *U. lactuca* is plentiful. *N. luetkeana* may be less common in their preferred habitat, which may explain why it was less dominant in the isotope results. Additionally, because isotopic analysis captures a longer time-integrated window of feeding activity (e.g., days) (Zanden et al., 2015) relative to the feeding trials which represent a snapshot in time, it is reasonable to expect the isotope results to show a more equal distribution among macrophytes (**Figure 4**).

Kelp can bioaccumulate in large quantities within seagrass meadows at certain times of the year (Wernberg et al., 2006; Krumhansl and Scheibling, 2012), and mesograzers may be able to respond to these pulses for their nutritional benefit, as demonstrated in the lab experiments. The *Z. marina* meadow examined here is surrounded by both *N. luetkeana* and *M. pyrifera* kelp forests (**Figure 1**) which culminate to large quantities of sea wrack biomass in the area (Wickham et al., 2020) and is thus likely available *via* detrital and POM pathways to mesograzers. Sediment isotopic analysis in this region has found kelp in sediment carbon pools (Prentice et al., 2019), indicating its accumulation in the not necessary meadow. *N. luetkeana* is an annual species that exhibits higher rates of productivity in the summer months (Maxell and Miller, 1996), when breakage and sloughing can produce the drift material found in seagrass beds. Major exports of kelp in the fall occur past the peak *S. naiadum* growing season, and may fill an important part of mesograzers fall and winter diets.

Examining the C:N ratios of macrophytes, macroalgae (*S. naiadum*, *U. lactuca*, and *N. luetkeana*) had lower C:N ratios, suggesting relatively higher palatability compared to *Z. marina* (**Table 1**). Given that *S. naiadum* epiphyte loads can be high (up to 5.1g dry weight per eelgrass shoot), our results indicate that *S. naiadum* is an important autochthonous contributor to mesograzer diets. Other nutritional properties are not captured in the C:N ratio that may make *N. luetkeana* a desirable food source, such as low levels of polyphenolic defense compounds (Steinberg, 1985; Pennings et al., 2000) or increased fatty acids or polysaccharides. More generally, *N. luetkeana* appears to be a preferred food choice for a variety of nearshore mesograzers, as seen in not necessary *Tegula funebralis* (Steinberg, 1985) (Steinberg, 1985), *Pugettia producta* (Dobkowski et al., 2017), *Idotea wosnesenskii* (Dethier et al., 2014), and *Strongylocentrotus droebachiensis* larvae (Feehan et al., 2018).

Although we did not look at it explicitly due to its relatively low biomass in the area, the giant kelp *M. pyrifera*, integrate into the food web at similar isotopic values to *N. luetkeana* (e.g, Monteray Bay δ13C ranging from -14.93 ± 0.52 ‰ to -20.54 ± 0.81‰, Drobnitch et al., 2018). Given the dominance of *N. luetkeana* in the study area (**Figure 1**) it is likely we captured a representative take on mesograzer diets, however examining temporal feed preferences of *M. pyrifera* alongside *N. luetkeana* would be a worthwhile, particularly because *M. pyrifera* the biomass available year-round.

While we did not examine periphyton (e.g., diatoms) as a potential food source it represents another ubiquitous and sometimes abundant food source that should be considered. Epiphytic microalgae may be a more important food source for gastropod mesograzers than to arthropod mesograzers, due to their low mobility (Doropoulos et al., 2009). This also may explain why *Z. marina* was found to be the dominant contribution to Lacuna snail diets (**Figure 4E**), as they may ingest surface layers of *Z. marina* tissue while scraping the blades for periphyton. Studies elsewhere have shown that these epiphytes can have similar δ^13^C values to seagrass, examples ranging from -11.3 ± 0.81‰ (Jaschinski et al., 2008) to −15 ± 1.5 ‰ (Mittermayr et al., 2014), whereas *Z. marina* can range from -9.64 ± 0.65‰ (Jaschinski et al., 2008) to -13.4 ± 3.3 ‰ (Mittermayr et al., 2014).

In other nearshore regions, allochthonous kelp subsidies has been found in the diets of seagrass meadow mesograzers. In Australia, the kelp *Ecklonia radiata* made a notable contribution to the diets of two seagrass-dwelling gastropods (Doropoulos et al., 2009), and gastropod species were found to assimilate isotopically-labeled δ^15^N kelp under both field and laboratory conditions (Hyndes et al., 2012). Further, *in-situ* addition of kelp to *Posidonia sinuosa* seagrass plots increased the densities and biomass of the gastropod *Strigosella lepidus* and shrimp (Cartraud et al., 2021). Finally, the trophic incorporation of kelp subsidies by seagrass dwelling species has been demonstrated at higher levels of the food web such as fish (Wernberg et al., 2006; Olson et al., 2019).

### 4.3 Trophic implications of kelp subsidies to eelgrass meadows

When allochthonous inputs are high, there is potential for the recipient ecosystem’s food web structure to be altered (Zuercher and Galloway, 2019). In seagrass ecosystems, the relationship between mesograzers and epiphytes is important for maintaining meadow health, as mesograzers prevent epiphytes from outcompeting seagrass for light and nutrients. Based on this typical seagrass trophic structure, if mesograzers were primarily consuming epiphytes, we would expect to observe an inverse relationship between mesograzer abundance and epiphyte biomass. A preliminary glance at the trophic structure in Choked Passage revealed a positive relationship between gammarid amphipods and *S. naiadum* (**Figure 5A**).

In addition to being a food source, *S. naiadum* may be providing habitat for some mesograzer species, supporting the positive relationship observed with gammarid amphipods. Epiphytes are known to add structural complexity to meadows which can increase mesograzer abundance and diversity (Viejo, 1999). We anecdotally observed amphipods exhibiting tube-building behavior within the *S. naiadum* epiphytes, and unpublished gammarid amphipod-*S. naiadum* feeding trials revealed very little consumption of *S. naiadum* and only a minor increase in mass lost with an increase in amphipod number. Thus, there may be a number of biotic and abiotic factors, including the availability of allochthonous food sources, that may be driving this positive mesograzer-epiphyte relationship. Moreover, gammarid and caprellid amphipod biomass was highest at transects next to kelp, suggesting potential edge effects from kelp - increasing food for mesograzers; or adding habitat from increased structural complexity (Olson et al., 2019). These results support bottom-up structuring in this system with allochthonous subsidies playing some role in shaping this ecosystem, however it may be minor compared to the environmental drivers.

Matched seasonal dynamics and bottom-up control of epiphytes and mesograzers may be also at play (Fong et al., 2000; O’Connor et al., 2022). High currents in the study area likely contribute to the persistence of *S. naiadum*, as the constant replenishment of water can supply plentiful nutrients to both seagrass and epiphytes, and maintains a low turbidity water column which reduces competition for light (O’Connor et al., 2022). Our inference of kelp's role in structuring the food web is also limited by the observational nature of our study data. *In situ* experimental addition or exclusion of kelp subsidies would add more insight on the causal effects of kelp on mesograzer-epiphyte relationships (e.g., Cartraud et al., 2021). The expectation of a negative relationship may be more applicable to interactions with smaller epiphytes or diatoms where a reduction in biomass could be reduced expeditiously by a similar abundance of mesograzers. A subsidy effect may further vary based on characteristics of the focal epiphyte (e.g., habitat-forming, nutritional quality, availability) and mesograzers (e.g., size, mobility, feeding behavior). Further research to decipher mechanisms of bottom-up drivers is needed to understand the full effect of kelp to seagrass food webs.

## 5 Conclusion

Seagrass meadows are highly productive ecosystems that not only export large quantities of biomass, but can also receive energy *via* allochthonous kelp from neighboring habitats. Our results suggest that inputs of allochthonous kelp are important to recipient *Z. marina* food webs through mesograzer consumption. Seagrass and kelp forests face concurrent challenges across their ranges; thus gaining a better understanding of the prevalence and magnitude of linkages among marine ecosystems is timely. Further characterizing the flows of allochthonous energy into and out of seagrass habitats can help us better understand their roles in climate change mitigation and habitat provisioning.

## Data availability statement

The original contributions presented in the study are included in the article/**Supplementary Material**, further inquiries can be directed to the corresponding author/s.

## Author contributions

AO, CP, MHL, FJ participated in sampling design. AO, CP, DV collected the data, processed samples. AO, CP, ZM analyzed data. AO, CP, and MHL wrote the manuscript, and all authors provided valuable feedback on the manuscript. All authors contributed to the article and approved the submitted version.

## Funding

Funding for this study was provided by the Tula Foundation and the University of Victoria.

## Acknowledgments

We thank the Tula Foundation for research support for the Hakai Institute’s Nearshore Ecology Program. Research support was given by the University of Victoria to AO and FJ. We thank Hakai Staff (Tristan Blaine, and Natasha Salter) and the UVic Fisheries Ecology and Conservation Lab for their role in data collection, the Hakai Oceanography Program for the temperature data from Pruth Bay, and Hakai Geospatial for providing a map of the study area.

## Conflict of interest

The authors declare that the research was conducted in the absence of any commercial or financial relationships that could be construed as a potential conflict of interest.

## Publisher’s note

All claims expressed in this article are solely those of the authors and do not necessarily represent those of their affiliated organizations, or those of the publisher, the editors and the reviewers. Any product that may be evaluated in this article, or claim that may be made by its manufacturer, is not guaranteed or endorsed by the publisher.
