# Supplementary material for: Grazing preference and isotopic contributions of kelp to Zostera marina mesograzers
Source: Front Plant Sci. 2022 Oct 13;13:991744. doi: 10.3389/fpls.2022.991744 (PMC9608150; doi:10.3389/fpls.2022.991744)
Supplement: Supplementary file 1 [file DataSheet_1.docx]

***Supplementary Material***

# **1** **Supplementary Figures**

**Supplementary Figure 1.** Top 5 m surface water temperatures from Pruth Bay, Canada in August 2015.


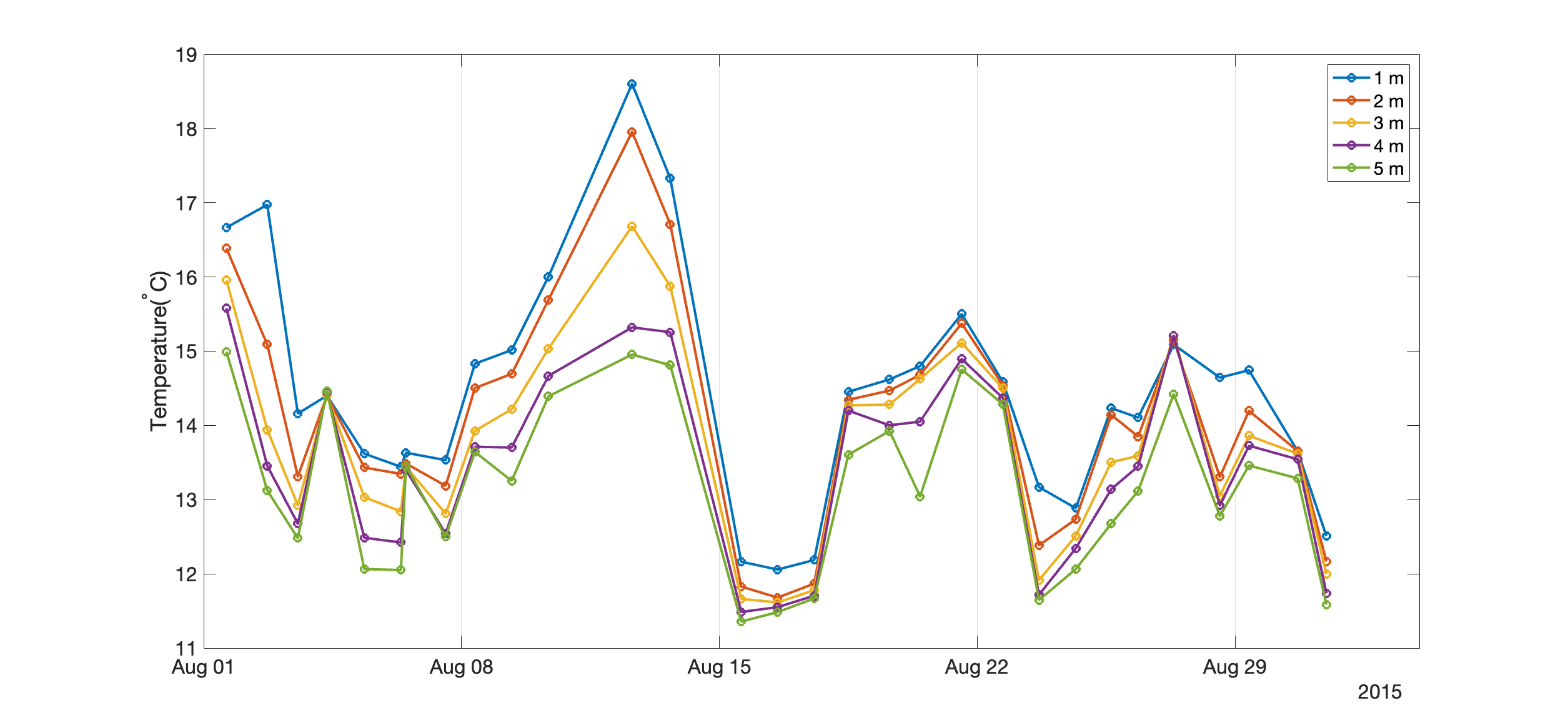


**Supplementary Figure 2.** Non-significant relationships from the gamma-hurdle model between *Smithora naiadum* and grazer biomass: (A) *Idotea resecata*, (B) caprellid amphipods, and (C) Lacuna snail biomass; and (D) meadow location and Lacuna snail biomass. Shaded blue indicates 95% confidence intervals.
